# Neuronal avalanches and time-frequency representations in stimulus-evoked activity

**DOI:** 10.1038/s41598-019-49788-5

**Published:** 2019-09-16

**Authors:** Oshrit Arviv, Abraham Goldstein, Oren Shriki

**Affiliations:** 1 Department of Cognitive and Brain Sciences, Ben-Gurion University of the Negev, Beer-Sheva, Israel; 2 The Inter-Faculty School for Brain Sciences, Zlotowski Center for Neuroscience, Ben-Gurion University of the Negev, Beer-Sheva, Israel; 3 The Leslie and Susan Gonda (Goldschmied) Multidisciplinary Brain Research Center, Bar-Ilan University, Ramat Gan, Israel; 4 Department of Psychology, Bar-Ilan University, Ramat Gan, Israel; 5 Department of Computer Science, Ben-Gurion University of the Negev, Beer-Sheva, Israel

**Keywords:** Dynamical systems, Neural circuits

## Abstract

Neuronal avalanches are a hallmark feature of critical dynamics in the brain. While the theoretical framework of a critical branching processes is generally accepted for describing avalanches during ongoing brain activity, there is a current debate about the corresponding dynamical description during stimulus-evoked activity. As the brain activity evoked by external stimuli considerably varies in magnitude across time, it is not clear whether the parameters that govern the neuronal avalanche analysis (a threshold or a temporal scale) should be adaptively altered to accommodate these changes. Here, the relationship between neuronal avalanches and time-frequency representations of stimulus-evoked activity is explored. We show that neuronal avalanche metrics, calculated under a fixed threshold and temporal scale, reflect genuine changes in the underlying dynamics. In particular, event-related synchronization and de-synchronization are shown to align with variations in the power-law exponents of avalanche size distributions and the branching parameter (neural gain), as well as in the spatio-temporal spreading of avalanches. Nonetheless, the scale-invariant behavior associated with avalanches is shown to be a robust feature of healthy brain dynamics, preserved across various periods of stimulus-evoked activity and frequency bands. Taken together, the combined results suggest that throughout stimulus-evoked responses the operating point of the dynamics may drift within an extended-critical-like region.

## Introduction

Criticality, a state found in complex systems situated at the edge of a phase transition, is marked by dynamical properties that lack any distinctive spatial or temporal scale. In the context of neuronal systems, critical phenomena have already gained substantial evidence^1–5^. Accordingly, power law distributions and scale-free correlations, observed at scales ranging from neuronal cultures to the whole brain, are very general characteristics of cortical activity, and therefore offer a unifying framework for the dynamical organization of this activity across scales^6–9^.

One of the most established hallmarks of scale-free critical dynamics are neuronal avalanches – propagating cascades of bursts of activity, whose sizes exhibit a power-law distribution. The neuronal avalanche analysis consists of identifying large positive and negative signal excursions beyond a threshold. The cascades are then identified by clustering these discrete supra-threshold events based on temporal proximity, thus, defining neuronal avalanches as periods of collective spatio-temporal organization, which are intercepted by prior- and post- relative silence. Since the pioneering research by Beggs and Plenz in 2003, many experimental works in several measurement modalities have found that neuronal avalanche analysis can be successfully explained using the framework of critical branching processes^3,10,11^. Accordingly, the critical exponent, α, of avalanche size distribution (P(s) ~ s^α^) was found to be approximately −3/2. Nonetheless, recently several publications pointed to the reported variations and possibly significant deviations from α = −3/2, questioning whether such measurements could reflect different critical exponents that potentially belong to universality classes of other distinct critical regimes^12–15^.

One interesting scenario of potential deviations from the critical branching process is during stimulus-evoked activity, *i*.*e*., in response to external sensory input^16–18^. Previous studies indicate that on long temporal scales (~1 sec) despite the presence of a sensory drive, the approximate description of a critical branching process holds, whereas on shorter temporal scales there are deviations from this description^16,17^. Specifically, in Arviv *et al*.^17^, we reported highly similar scale-free avalanche size distributions for stimulus-evoked activity and ongoing resting state over ~1 sec intervals. Yet, stimulus-evoked activity was characterized by a tendency for larger size avalanches and temporary deviations from the predictions of a critical branching process. This suggests that sensory adaptation and other compensating mechanisms may be involved in maintaining criticality over long temporal scales. In a recent article, Yu *et al*.^19^ suggested that the proximity to a critical branching process is maintained all throughout stimulus-evoked activity (even on relatively short temporal scales of 100 or 400 msec), much like in the mean-field description of ongoing brain activity. According to this publication, stimulus-evoked activity should be divided to periods or windows which are associated with distinct supra-threshold event rate. The authors claim that the parameters of the neuronal avalanche analysis should be adapted to the average event rate characterizing each period. By doing so, the authors were able to maintain similar distributions and critical exponent, α, for all stimulus-evoked periods.

Stimulus-evoked responses are characterized by temporal modulations in the frequency domain^20^. Therefore, the relationship between neuronal avalanches and spectral changes can be straightforwardly investigated during stimulus-evoked activity. In addition, any potential deviations from criticality during stimulus evoked activity may be interconnected to variations in the magnitude and spectral content of recorded activity. Here, we take a closer look at the underlying dynamics of different periods of stimulus-evoked activity by applying spectral decompositions as well as focusing on substantially shorter time intervals (80 ms). Obtaining time-frequency representations of evoked-responses can shed light on the evolving dynamics and the networks involved during such periods. Accordingly, we suggest that (1) neuronal avalanche analysis can reflect similar dynamical and topographical characteristics as time-frequency representations. Moreover, neuronal avalanche analysis does not rely on time intervals, other than the time of interest (e.g., base-line correction), in order to capture spatiotemporal dynamics. (2) stimulus-evoked dynamics are better reflected by avoiding simple compensating (adaptive) changes in the parameters of the neuronal avalanche analysis that aim to mimic fluctuations in event rate. Rather, some of the changes in stimulus-evoked dynamics can be represented by allowing critical exponents to freely change. (3) scale-free behavior is a relatively robust feature of cortical dynamics, preserved during various periods of stimulus-evoked responses and frequency bands. We suggest that changes in the underlying dynamics of stimulus-evoked responses may shift the operating point of the neural system within an extended critical-like region (Griffiths phase)^21–23^. This may cause changes in the corresponding power-law exponents, while maintaining scale-free statistics.

## Results

Using the framework of neuronal avalanches, both stimulus-evoked and resting state brain activities are examined. Particularly, in order to assess the sensitivity of the neuronal avalanche analysis to evoked responses and its relations to temporal and spectral features, we analyzed MEG data collected from a group of subjects (n = 21, age = 22.6 ± 3.0 years) preforming a visual task of face perception^17^.

### Neuronal avalanche analysis differentiates between stimulus-evoked and resting-state activities over short time intervals

**Figure 1:**
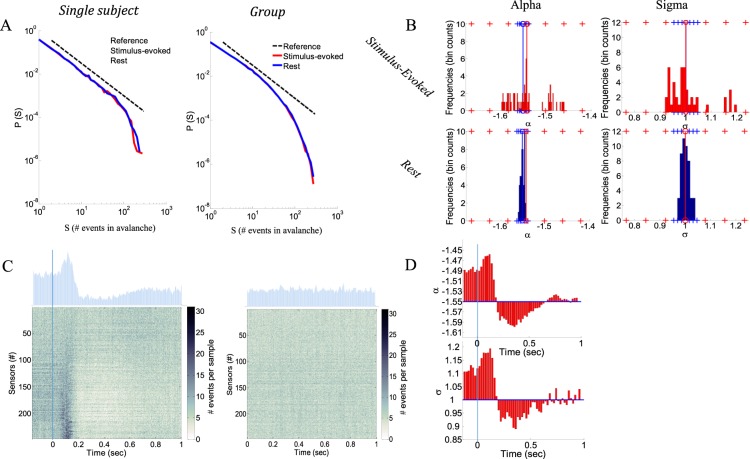
Neuronal avalanche analyses of stimulus evoked and resting state activities. (**A**) Avalanche size distributions of a single subject (right panel) and of the group of subjects (left panel). Notably, at both the single subject and the group, the distributions of stimulus evoked and rest overlap. Additionally, due to between-subject variability at the cut-off, the group distribution (across all subjects) demonstrate a more curved and gradual decline in the slope. Estimations of best-fit parameters rely more heavily on the higher probability (small avalanches) limit (see Methods). (**B**) Calculating the power law exponents, α, and the branching parameter, σ, for avalanches accumulated during 80 msec intervals resulted in more disperse histograms for stimulus evoked activity versus rest. Blue and red vertical lines indicate the averages of the rest and stimulus evoked histograms, respectively. Markers of plus sign indicate SD = 1, 2, 3 from the mean. (**C**) Grand supra-threshold event rasters of stimulus-evoked (left panel) and rest (right panel) and PSTHs (above rasters). An event is marked by a dot at the relevant sensor and time point. The color of the dot is the number of events summed across all trials and subjects. (**D**) Time-dependent stimulus-evoked histograms of α and σ relative to the mean over the resting state (the y-axis ticks indicate the corresponding values of stimulus evoked activity) reveal a similar trend as the grand raster (right panel in C).

Figure 1A (left panel) displays avalanche size distributions for a single subject at stimulus-evoked and resting-state activity. The distributions obey power law behavior, P(*s*) ~ *s*^α^, for both states (α = −1.54, at stimulus-evoked and rest). The cutoff of the power law was previously shown to be a function of the size of the sensor array, and thus relating to the finite spatial limits of the system^11,24^. The branching parameter, σ, which represents the neural gain and denotes the ratio between the numbers of elevated activity events in consecutive time steps, is also as expected for critical systems (σ = 1.00 and 1.01 for evoked and rest, respectively), that is, one local synchronous group (ancestor) triggers on average one other local synchronous group (descendant).

In Arviv *et al*.^17^, we showed that the avalanche size distributions of both cognitive states for relatively long segments (1 sec trials) closely resemble each other for each individual subject, and that their characteristics align with near-critical dynamics. In view of the consistency across subjects, we introduced a group analysis for the neuronal avalanche approach, accumulating cascades from all subjects. Indeed, for the relatively long segments (1 sec trials), Fig. 1A (right panel) group analysis demonstrates that avalanche size distributions of both cognitive states closely resemble each other (in fact the two distributions nearly overlap) (α = −1.52, σ = 1.10 and 1.08 for evoked and rest, respectively). Notably, the group size distributions (Fig. 1A, right panel, and all displayed distributions onwards) appear to be curved at the cutoff region. Combining avalanches collected from different subjects to a joint group distribution smooths the cutoff, since for each subject the cutoff occurs at different typical avalanche size (due to variations of head size versus MEG helmet or in the dynamics itself^25^). Therefore, group distributions are expected to demonstrate a less defined cutoff and a more gradual decline in the slope compared to individual distributions. Nevertheless, the improved sample sizes justify the group approach, despite the between-subject variability. The maximum likelihood estimations, which were applied directly to the samples of avalanche sizes, and not to the distributions, provide increased sensitivity to sizes of higher probability (small avalanches) (see Methods). Testing the power-law hypothesis, the group distributions at both cognitive states remained in favor of a power-law model, as opposed to alternative hypotheses^17^. When Comparing single-parameter distribution models, the calculated log of the likelihood-ratio (LLR) demonstrated a significantly higher likelihood of a power law compared to an exponential function [LLR > 8 * 10^4^, p-values as small as the precision limit (p < 10^−324^)]. When comparing distribution models with two parameters, the LLR was higher for an exponentially truncated power law compared to lognormal and stretched exponential distributions (LLR_truncPL_Lognormal_ > 10^3^, p < 10^−154^, LLR_stretched_Lognormal_ > 4 * 10^2^, p < 10^−324^, LLR_truncPL_stretched_ > 8 * 10^2^, p < 10^−84^).

Here, the group analysis approach enabled us to focus on shorter time intervals, as the cascades accumulated across subjects resulted in sufficient sampling and statistics. Accordingly, group analysis was applied for 80 msec time intervals (sliding windows with a 20 msec overlap). The cascades collected obey power law distributions (α = −1.55 ± 0.04, α = −1.552 ± 0.004, and σ = 1.00 ± 0.08, σ = 1.00 ± 0.02 for evoked and rest, respectively). Figure 1B demonstrates the histograms of α and σ calculated for the 80 msec intervals. Comparing the histograms obtained from stimulus evoked to resting state, it is clear that histograms obtained for rest are very narrow around the mean, whereas the histograms of the stimulus-evoked state are more dispersed (Fig. 1B).

**Figure 2:**
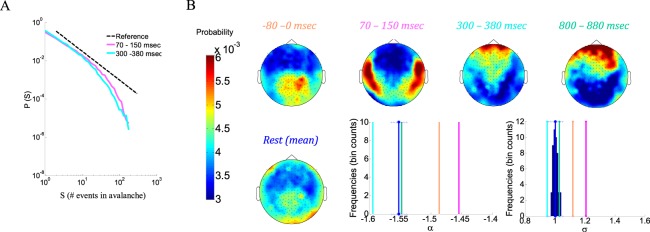
Neuronal avalanche analyses of exemplar intervals of stimulus evoked activity. (**A**) Avalanche size distributions, and (**B**) topographies of sensors’ probabilities to participate in avalanches, for particular time intervals (offset from t = 0, stimulus onset). Vertical lines indicate the specific α and σ of each particular interval corresponding to the rest histogram and its mean.

The first step of the neuronal avalanche analysis consists of discretizing the continuous electromagnetic signals by identifying supra-threshold events. Here, a fixed threshold of ±3 sd was applied. Subsequently, cascades were identified in each of the electromagnetic traces (*i*.*e*., per subject and of specific trial), by clustering the discrete events based on temporal proximity. The time bin for clustering was set here to be Δt = 2.95 msec (for details see Methods). Figure 1C, adopted from Arviv *et al*.^17^, portrays all accumulated events across all subjects and trials in raster plots, while on top of each raster plot are the peristimulus time histograms (PSTHs), a sample-wise summation of all events over sensors. This figure demonstrates for stimulus-evoked state (left panel), a change in the frequency of events across time (locked to stimulus onset, t = 0), which is not seen for spontaneous resting state: following stimulus onset, there is a significant peak, followed by a decrease and slow rise till average event frequency. In Figs 1C,D and 2B, we also included pre-stimulus intervals (prior to the 1 sec trials), characterized by an elevated frequency of identified events compared to the average and to rest. In the neuronal avalanche analysis, cascades are collected for each trace (*e*.*g*., trial) separately. Nonetheless, the α and σ calculated for 80 msec intervals now plotted as a function of time, and relative to the means associated with resting state (Fig. 1D, as opposed to the top row of Fig. 1B), follow the same trend seen in event rate. Yet, although the obtained values considerably deviate from those obtained in resting state, all size distributions follow a scale-free behavior [a significantly higher likelihood of a power law compared to an exponential function: LLR > 5 * 10^3^, p < 10^−75^, p < 10^−93^, for evoked and rest, respectively, as well as a significantly higher likelihood of an exponentially truncated power law compared to lognormal and stretched exponential distributions for both evoked and rest: LLR_truncPL_Lognormal_ > 4 * 10^1^, p < 10^−3^, LLR_stretched_Lognormal_ > 10^1^, p < 10^−21^, LLR_truncPL_stretched_ > 10^1^, (p < 10^−3^, with the exception of two segments of marginal significance p < 0.1, and two non-significant segments. All 4 segments are from evoked activity and relate to low event rates, and thus with a relatively small sample of avalanches)]. Figure 2A, represents neuronal avalanche size distributions of post-stimulus evoked response at 70–150 msec (α = −1.45, σ = 1.21) and at 300–380 msec (α = −1.60, σ = 0.95). Topographies displaying probabilities of each sensor to participate in avalanches during representative time intervals (summing across all sensors will give 1) are presented in Fig. 2B: pre-stimulus (−80–0 msec) (α = −1.49, σ = 1.11), post-stimulus evoked response (70–150 msec) (α = −1.45, σ = 1.21), post-stimulus decrease (300–380 msec) (α = −1.60, σ = 0.95), post-stimulus return to average (800–880 msec) (α = −1.55, σ = 1.02), and mean topography of all rest intervals (α = −1.552 ± 0.004, σ = 1.00 ± 0.02); the associated α and σ are represented alongside the rest histograms, with only the 800 msec interval not deviating by more than 3 SD from the mean of the rest distributions. Markedly, the same tendency of results was obtained, when avalanches assigned to each 80 msec interval were not limited by the interval limits [*i*.*e*., the analysis was ran over 1 sec segments and all avalanches partially contained within each specific 80 msec interval were collected, overpassing the hindrance for large avalanches [rest: α = −1.494 ± 0.005, σ = 1.12 ± 0.01; evoked: α = −1.43, σ = 1.26, α = −1.41, σ = 1.30, α = −1.55, σ = 1.04, α = −1.49, σ = 1.11, (Testing model likelihood – power law is better than exponential function: rest: LLR > 8 * 10^3^, p < 10^−122^; evoked: LLR > 7 * 10^3^, p < 10^−104^ and exponentially truncated power law is better than lognormal and stretched exponential distributions: rest: LLR_truncPL_Lognormal_ > 6 * 10^1^, p < 10^−5^, LLR_stretched_Lognormal_ > 10^1^, p < 10^−27^, LLR_truncPL_stretched_ > 10^1^, p < 10^−2^; evoked: LLR_truncPL_Lognormal_ > 6 * 10^1^, p < 10^−4^, LLR_stretched_Lognormal_ > 3 * 10^2^, p < 10^−26^, LLR_truncPL_stretched_ > 2 * 10^1^, p < 5 * 10^−2^)].

### Trial-based time-frequency decomposition and neuronal avalanche analysis exhibit similar dynamical and topographical characteristics

**Figure 3:**
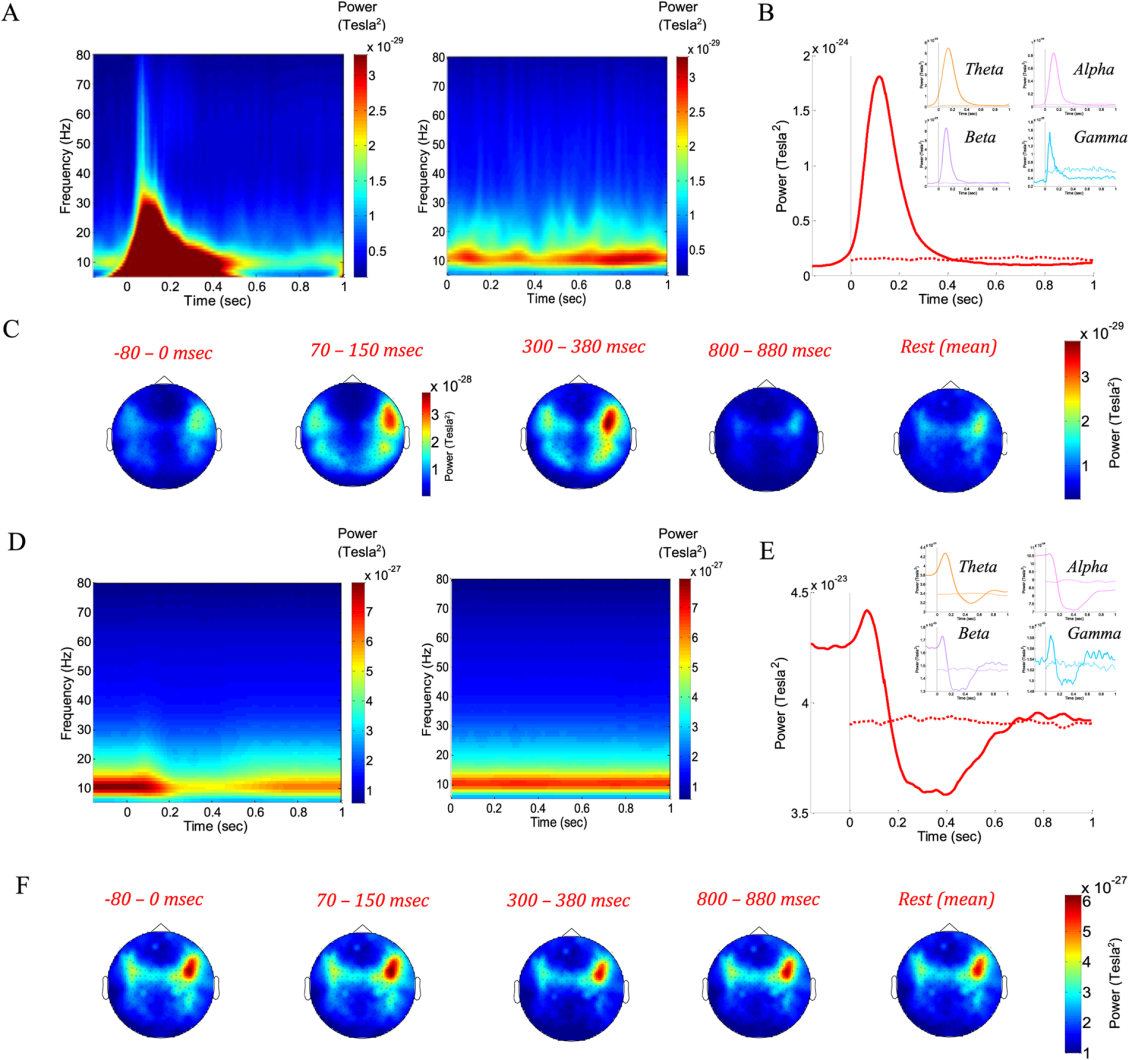
Time-frequency representations of rest and stimulus-evoked activities. “ERF time-frequency power” (**A**) and “total power” spectra across time (**D**) averaged over sensors (stimulus-evoked, left panel; rest, right panel). (**B**,**E**) Temporal profile of the summation over frequencies (1–80 Hz) and sensors of the relevant power-spectra across time (insets: theta 4–8 Hz: orange; alpha 8–13 Hz: pink; beta 13–30 Hz: violet; gamma 30–80 Hz: light blue; solid line – stimulus-evoked, broken line - rest). (**C**,**F**) Topographies of the summation over frequencies (1–80 Hz) and time interval (indicated above) of the relevant power-spectra across time. In (**C**) the colorbar of the 70–150 msec interval (corresponding to the most pronounced evoked-response) is different from those of the other topographies. The “total power”, averaging the time-frequency decomposition obtained for each trial (aligned to t = 0), reveals a temporal profile similar to the profile obtained from neuronal avalanche analysis.

Studying Event-Related Fields (ERFs) of these datasets emphasizes different aspects of the dynamics, demonstrating ERF components associated with face processing^17^. Here, we analyzed “ERF time-frequency power” for the frequency range consisting of the theta to gamma (4–80 Hz) frequency bands. Figure 3A–C depict time-frequency matrix averaged over all sensors (A), summation across sensors and frequencies (B), and summation across time and frequencies (C). As expected, the ERF time-frequency power representations do not have well-defined frequency components, due to mixing in the time domain during averaging and to the violation of stationarity during ERFs^20^. Nonetheless, the evoked response is limited to ~300 msec from stimulus onset and has similar appearance across frequency bands. The ERF baseline activity contains only diminutive variability in all frequency bands due to averaging across time. In contrast, analyzing “total power”, comprising both phase-locked and non-phase-locked activity, revealed alongside a discernible evoked response peak (at theta, beta and gamma frequency bands), an induced pre-event synchronization (at theta, alpha and beta frequency bands) and a post-event desynchronization (at all frequencies) (Fig. 3D left panel and E). While the “ERF time-frequency power” have no pre- and post- activation responses corresponding to the dynamical profile revealed by avalanche analysis (Fig. 1C,D), the total power shows a similar profile to the avalanche analysis. Notably, as expected, the “total power” has a narrower and more confined frequency structure.

**Figure 4:**
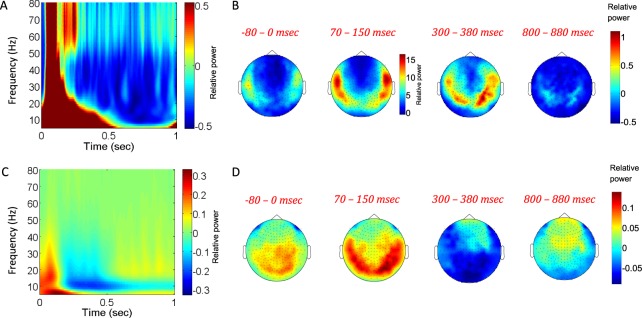
Relative time-frequency representations of stimulus-evoked activity versus rest. (**A**,**B**) and (**C**,**D**) correspond to (Fig. 3A,C,D,F), respectively, when subtracting from the stimulus-evoked spectra the rest spectra and dividing by rest. This gives the relative changes in the power-spectra across time. In (**B**), the colorbar of the 70–150 msec interval (corresponding to the most pronounced evoked-response) is different from those of the other topographies. Overall, the relative “total power” (**D**) reveals similar topographies to those obtained by avalanches (Fig. 2B).

Comprising the “total power”, both the ERFs and induced event-related synchronization/de-synchronization (ERS and ERD) are task-related, time-locked to stimulus-onset (time = 0), yet the ERS/D are not phase-locked to time = 0. However, another component of “total power” is the background activity which is not event-related and has no particular relations to time = 0. Resting state activity is by definition unrelated to a task, and therefore neither phase-locked nor time-locked to any particular event. Accordingly, it is potentially similar to the background activity mentioned above and can still be measured by time-frequency power (Fig. 3A,C,D,F left panels). Figure 4 shows the relative “ERF time-frequency power” and “total power” (spectrograms and topographies of means across indicated time intervals). The relative time-frequency representations are obtained by subtracting from the evoked responses the corresponding resting state and normalizing by the resting state). The obtained topographies for “relative total power” demonstrate a clear similarity to topographies obtained from the avalanche analysis by the naïve approach of calculating sensors’ probabilities to participate in an avalanche (Figs 2B and 4D). Notably, the avalanche analysis invoked a thresholding operation on the inspected time intervals, whereas the “total power” approach relies on baseline correction based on entirely separated time intervals.

### A scale-free behavior is maintained for short time intervals of stimulus-evoked activity, while (σ, α phase plots display a similar yet shifted behavior

**Figure 5:**
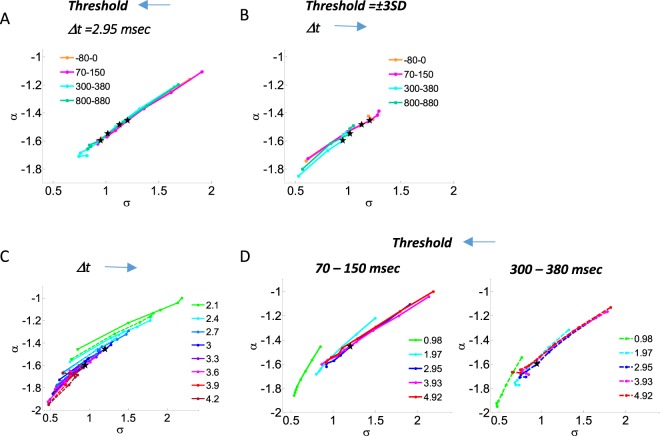
The influence of changing threshold and bin durations on the (σ, α) phase plots during stimulus-evoked activity. (**A**,**B**) Phase plots for specific 80 msec intervals (t = 0, stimulus onset), when in (**A**) Changing the threshold, while holding a fix bin duration (2.95 msec) and in (**B**) Changing bin duration, while holding a fix threshold (±3 SD). (**C**,**D**) While changing both threshold and bin durations: (**C**) displays multiple curves, each of a fix threshold, for the 70–150 msec (solid line) and the 300–380 msec (dashed line). This two time intervals show the stronger difference among the tested stimulus-evoked intervals. (**D**) Displays multiple curves, each of a fix bin duration for the 70–150 msec (solid line, left panel) and the 300–380 msec (dashed line, right panel). In all phase plots a black star indicates the (σ, α) obtained for the bin duration of 2.95 msec and threshold of 3 SD, which are the parameter values used for the prior avalanche analyses in this manuscript. These values for bin duration and threshold are those utilized to obtain (σ, α) at close proximity to the (σ = 1, α = −1.5) predicted from critical dynamics theory.

In the avalanche analysis, the fixed threshold (±3 sd) and bin duration (Δt = 2.95 msec) were set in accordance with predicted values for critical branching dynamics of a power law exponent α ≈ −1.5, and a branching parameter σ ≈ 1 during 1 sec segments of resting state as well as stimulus-evoked state. Zooming on particular intervals in stimulus-evoked state (Figs 1D and 2), characterized by a different event rate (Fig. 1C), resulted in variations in α and σ. Nonetheless, changing the threshold and/or bin duration, leads to different values for α and σ. Figure 5A,C display phase plots of α as a function of σ, while altering either the threshold or the bin duration (respectively). The different stimulus-evoked intervals show a very similar monotonic behavior, only the curves are slightly shifted from one another. As the threshold increases (Fig. 5A: moving on the curve in the left direction), the number of identified discrete events decreases and there is a monotonic reduction in avalanche sizes. As the bin duration increases (Fig. 5B: moving on the curve in the right direction), by definition the required quiescent periods pre- and post- cascade increase, resulting in the merging of what in shorter bin duration would have counted as separate cascades. Thus, there is a monotonic increase in avalanche sizes. Examining the variation in both threshold and bin duration (Fig. 5C,D) for the stimulus-evoked intervals demonstrating the largest difference, 70–150 msec and 300–380 msec (Fig. 2A), showed there is a consistent shift between (σ, α) phase plots for all thresholds and bin durations, while maintaining a close similarity in behavior. Testing the model likelihood for all four examined stimulus-evoked intervals while changing both threshold and bin duration showed a higher likelihood to a power-law compared to an exponential function (LLR(min, max) = [2 * 10^2^, 7 * 10^4^], p(min, max) = [10^−324^, 10^−4^]), as well as a significantly higher likelihood to an exponentially truncated power law compared to lognormal and stretched exponential distributions [LLR_truncPL_Lognormal_ (min, max) = [2, 10^3^], (p(min, max) = [10^−324^, 10^−2^], excluding the interval 800–880 msec: at the highest threshold of 4.2 SD when Δt is equal or larger to 2.95 msec, and the interval 300–380 msec: at thresholds larger than 3.3 SD when Δt equal or larger than 2.95 msec, (the lower thresholds 3.3 and 3.6 SD or the smaller Δt 2.95 msec are related to marginally significant p-values (p < 0.1), higher thresholds while at larger Δt are non-significant), LLR_stretched_Lognormal_ (min, max) = [2, 5 * 10^2^], p(min, max) = [10^−324^, 4 * 10^−2^], LLR_truncPL_stretched_ (min, max) = [2, 10^3^], (p(min, max) = [10^−324^, 5 * 10^−2^], excluding the interval −80–0 msec with marginal significance p < 0.1 at highest threshold when Δt is the largest, and the interval 800–880 msec at highest threshold, 4.2 SD, while Δt larger than 0.98 msec (*i*.*e*., marginally significant at Δt 1.97 msec, and non-significance at larger Δt). For the interval 300–380 msec substantial parameter combinations correlated with a non-significant p-values: all thresholds equal or above 2.7 SD at Δt equal or above 2.95 msec, while one of these combinations resulted in negative LLR (LLR = −0.5 and p = 0.96))]. Overall power-law (one parameter) and exponentially truncated power-law (two parameters) are significantly better models for the majority of examined thresholds and Δt-s during periods of stimulus-evoked responses. Moreover, all the exceptions in the evaluated significance correspond with relatively small sample of avalanches, due to combinations of higher thresholds, larger Δt, and periods related to lower event rate. Additionally, the combinations of higher thresholds and larger Δt-s also correspond to deviations in the monotonic behavior of the curves in the (σ, α) phase plots. Thus, the lower number of identified avalanches may have resulted in less reliable estimations of σ and α at the particular (threshold, Δt) combinations.

### A scale-free behavior is maintained for a range of thresholds and bin durations for frequency-band specific activities

**Figure 6:**
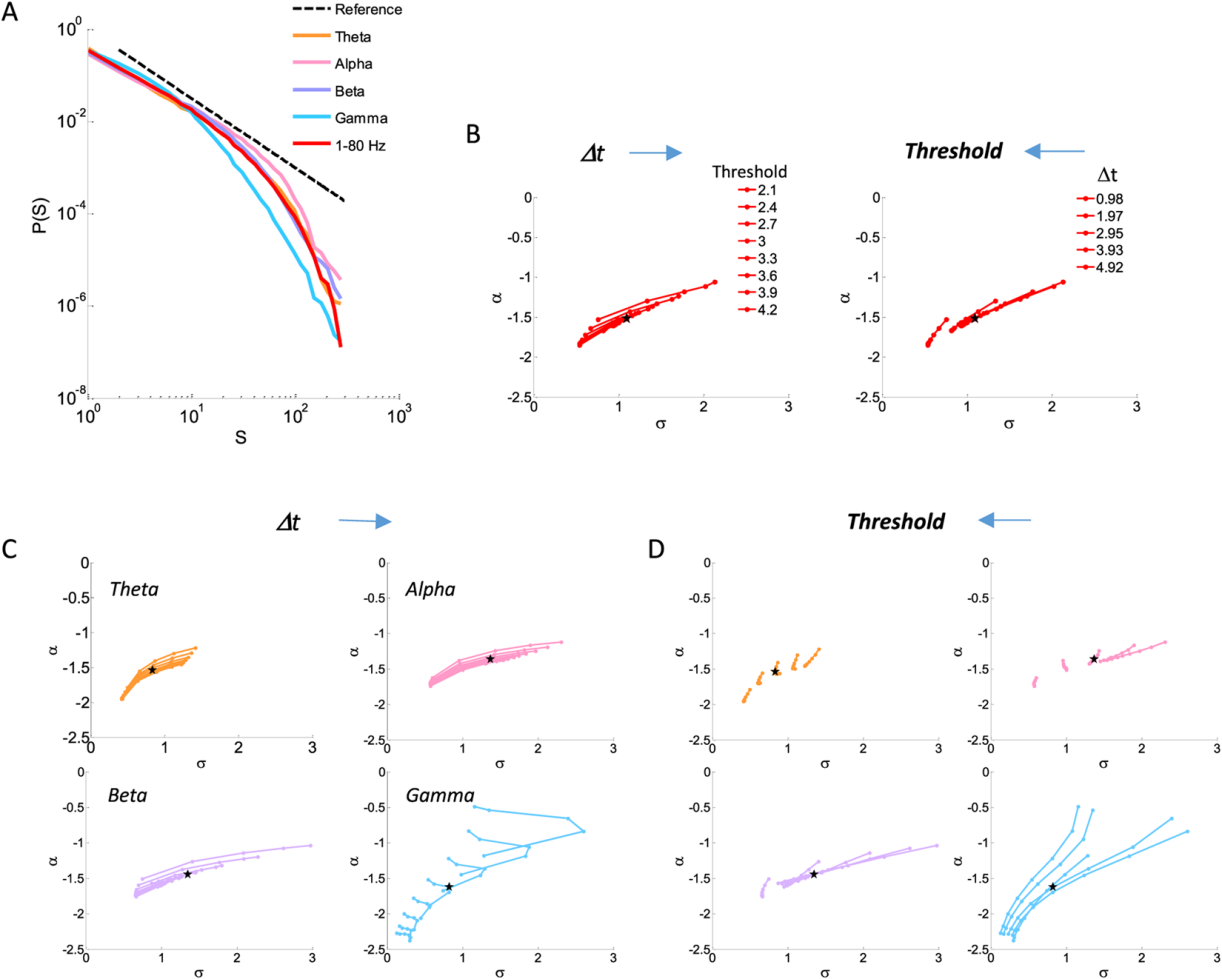
A scale-free behavior of each frequency band, excluding gamma, at different thresholds and bin durations. (**A**) Avalanche size distributions at all inspected frequency bands. (B, left panel) and (**C**) display in the (σ, α) phase plots, multiple curves at a fix threshold, while (B, right panel) and (**D**) display multiple curves at a fix bin duration. In all phase plots a black star indicates the (σ, α) obtained for the bin duration of 2.95 msec and threshold of ±3 SD.

Avalanche analysis can be applied to each frequency band separately. Using the parameters of fixed threshold (±3 sd) and bin duration (Δt = 2.95 msec), revealed a scale-free behavior in all inspected frequency bands, excluding the gamma band, for resting state and stimulus-evoked activity, accumulated across 1 sec epochs and subjects [frequency band (σ, α) evoked/rest: theta (−1.54, 0.84)/(−1.56, 0.82); alpha (−1.36, 1.37)/(−1.31,1.50); beta (−1.45, 1.35)/(−1.47, 1.32); gamma (−1.62, 0.83)/(−1.62, 0.82); while for 1–80 Hz (−1.52, 1.10)/(−1.52, 1.08)] (a significantly higher likelihood of a power law compared to an exponential function: LLR > 3 × 10^4^, p < 10^−180^, for all frequency bands at both evoked and rest, as well as a significantly higher likelihood of an exponentially truncated power law compared to lognormal and stretched exponential distributions: LLR_truncPL_Lognormal_ > 8 * 10^2^, p < 10^−153^, LLR_stretched_Lognormal_ > 2 * 10^2^, p < 10^−120^, LLR_truncPL_stretched_ > 4 * 10^2^, p < 10^−86^, for theta, alpha, beta frequency bands at both evoked and rest. Gamma band demonstrated a higher likelihood for the lognormal and stretched exponential than the exponentially truncated power law: LLR_truncPL_Lognormal_ < −3 * 10^3^, p < 10^−172^, LLR_truncPL_stretched_ < −3 * 10^3^, p < 10^−324^). Notably, stimulus-evoked and rest, demonstrate similar (σ, α) for each frequency band, yet, with a slight tendency for larger avalanches in the alpha frequency band during rest versus stimulus-evoked and an opposite even more minute tendency in the theta and beta frequency bands. For example, Fig. 6 demonstrates, the results for stimulus-evoked activity, when applying avalanche analysis on each frequency band (similar results for rest are not shown). Avalanche size distributions at (Δt = 2.95 msec, threshold = ±3 sd) are displayed at Fig. 6A. Changing the threshold and/or bin duration revealed that the scale-free behavior for the theta, alpha and beta frequency bands as well as for the broad band signal is maintained for a wide range of (threshold, Δt) values at both evoked and rest (a significantly higher likelihood of a power law compared to an exponential function: theta: LLR(min, max) = [3 * 10^3^, 5 * 10^5^], p(min, max) = [10^−324^, 10^−49^], alpha: LLR(min, max) = [10^3^, 9 * 10^5^], p(min, max) = [10^−324^, 10^−19^], beta: LLR(min, max) = [3 * 10^3^, 10^6^], p(min, max) = [10^−324^, 10^−34^], broad: LLR(min, max) = [5 * 10^3^, 8 * 10^5^], p(min, max) = [10^−324^, 10^−81^], as well as a significantly higher likelihood of an exponentially truncated power law compared to lognormal and stretched exponential distributions: theta: LLR_truncPL_Lognormal_ (min, max) = [5 * 10^1^, 8 * 10^3^], p(min, max) = [10^−324^, 10^−2^], LLR_stretched_Lognormal_ (min, max) = [3 * 10^1^, 10^4^], p(min, max) = [10^−324^, 9 * 10^−19^], LLR_truncPL_stretched_ (min, max) = [4 * 10^2^, 4 * 10^3^], p(min, max) = [10^−324^, 2 * 10^−2^] excluding smallest Δt at all thresholds which demonstrated a significantly higher likelihood to a stretched exponential distribution, alpha: LLR_truncPL_Lognormal_ (min, max) = [2 * 10^2^, 2 * 10^4^], p(min, max) = [10^−324^, 10^−20^], LLR_stretched_Lognormal_ (min, max) = [9 * 10^1^, 10^4^], p(min, max) = [10^−324^, 2 * 10^−55^], LLR_truncPL_stretched_ (min, max) = [6 * 10^1^, 6 * 10^3^], p(min, max) = [10^−324^, 10^−5^], beta: LLR_truncPL_Lognormal_ (min, max) = [10^2^, 2 * 10^4^], p(min, max) = [10^−324^, 9 * 10^−7^], LLR_stretched_Lognormal_ (min, max) = [8 * 10^1^, 8 * 10^3^], p(min, max) = [10^−324^, 5 * 10^−39^], LLR_truncPL_stretched_ (min, max) = [3 * 10^1^, 10^4^], p(min, max) = [10^−324^, 10^−2^] excluding largest Δt at lower thresholds (2.1–2.7 SD) which demonstrated a significantly higher likelihood to a stretched exponential distribution, as well as non-significant likelihood at the highest threshold, broad: LLR_truncPL_Lognormal_ (min, max) = [8 * 10^1^, 10^4^], p(min, max) = [10^−324^, 9 * 10^−8^], LLR_stretched_Lognormal_ (min, max) = [5 * 10^1^, 7 * 10^3^], p(min, max) = [10^−324^, 8 * 10^−37^], LLR_truncPL_stretched_ (min, max) = [3 * 10^1^, 8 * 10^3^], p(min, max) = [10^−324^, 8 * 10^−3^]). Gamma band demonstrated a non-consistent likelihood trend for variating combinations of (threshold, Δt), at large rejecting a scale-free behavior at this frequency band. Therefore, the estimated power-law exponent at this range of frequencies can only be regarded as a linear approximation at log-log coordinate system. Notably, the α(σ) curves for the gamma band at the examined (threshold, Δt) values, showed unusual behaviors compared to other frequency bands (Fig. 6C,D) and with a particular non-monotonicity with the increment of Δt (Fig. 6C). Across all frequency bands, a very similar trend was found for resting state as in stimulus evoked activity (*graphs not shown*).

## Discussion

Neuronal avalanches are manifestations of collective organization of activity, which naturally occur in the cortex. Many studies have demonstrated that spontaneous neuronal activity can be modeled with a good approximation by a critical branching process^1^. This study sheds light on the relations between neuronal avalanches and time frequency representations, while at the same time addresses the validity of the branching process model in describing stimulus-evoked activity.

Examining the time-frequency decompositions of stimulus-evoked activity and resting state reveals similarities between trial-based decompositions and avalanche analyses. Accordingly, both task related induced- and evoked- activities are sampled by the thresholding operation and the aggregation of the identified discrete events into avalanches. The temporal profile of time-locked spectral decomposition, which relies on phase-locked as well as non-phase locked activities, aligns with the temporal behavior revealed by the power-law exponent and branching parameter (Figs 1D and 3E). Thus, the fluctuations in metrics following the critical state dynamics capture similar properties as the additive ERS/D and ERFs. Markedly, similar topographies are obtained from the spectral and the avalanche analyses, yet only when the “total power” spectral decomposition is baseline-corrected by the resting-state decomposition (Figs 2B and 4D). This suggests a couple of things: one, that given that neuronal avalanches successfully capture the functional connectivity of neuronal networks^26^, neuronal avalanches can give a valid description of spatial spreading of activity without relying on any information taken outside of the studied time-interval – hence neuronal avalanches have an advantage over spectral description; two, that ongoing background activity (not task-related) has similar characteristics during rest as during stimulus-evoked responses, and therefore, subtracting this background activity does not hide the identity of task-related brain sites involved. It is worthwhile to remark that this does not exclusively support or discard whether evoked responses are additive to ongoing signals or are the result of frequency-dependent phase reset and alignment^20,27^, as the last can result in synchronizations that give “more of the same” activations, which will also survive thresholding.

Stimulus-evoked responses are associated with changes in band-limited activity, and in the 1/f component^28^, thus representing an extensive interplay that comes about in the measured signals. As was shown in Fig. 6, band-limited activities, as well as the broad-band activity, exhibit power-law cascade size distributions over a range of (threshold, Δt) combinations at stimulus-evoked (as well as at resting state, *data not shown*), indicative of a scale-free behavior. Specifically, the gamma band demonstrated at large a non-scale free behavior. The gamma band has a relatively broad frequency range, which consists of fast oscillations. Accordingly, these results could be the reflection of neurophysiological processes and/or compelled by the constraints of the applied signal processing (see Methods).

Another important feature is that stimulus-evoked activity is characterized by consistent variations in event rate (Fig. 1C). The fact that changes in ERFs and ERS/D (Fig. 3E) are tied to functional properties^29–32^, implies that the corresponding changes in event rate are also tied to functional aspects. Utilizing a fixed threshold (calculated relative to the mean activity over extended periods) highlights these changes (Figs 2 and 4C,D). Markedly, the interlinked parameters that govern the avalanche analyses (threshold and Δt), have an assortment of values in which they will enable detecting the same type of phenomenon, when shifts within this range will only affect the sensitivity of detecting events (“noise” level) – threshold; and the aggregation of events into cascades (joining events into a single cascade or isolating events into separate cascades) – Δt. As in rest, the examined time intervals of stimulus-evoked activity, for a range of (threshold, Δt) combinations, demonstrated scale-free avalanche size distributions. Additionally, when varying the thresholds or bin durations (Fig. 5), stimulus-evoked periods associated with different event rates resulted in (σ, α) curves of similar shape, yet shifted. This suggests similarity in dynamical regimes.

In this study, the neuronal avalanche analysis was applied without modifying the threshold or Δt as a function of event rate. The obtained results demonstrate that neuronal avalanches capture genuine changes in the underlying dynamics. Specially, that the changes found in power-law exponents correlate with the synchronization and desynchronization of the recorded activity, which in turn may reflect changes in the excitation-inhibition balance of the neuronal system^33,34^. Notably, Yu *et al*.^19^ suggested that periods associated with distinct event rates should be analyzed with adaptive time bins of varying duration Δt - set to the inverse of the average rate over each period, or alternatively by an adaptive threshold which can be the mean amplitude ± n · sd over each period (or over consecutive windows). In other words, they propose to adapt the analysis parameters to task- or event- related changes in rate. This enabled the authors to obtain similar distributions and power-law exponent, α, for all periods. Indeed, the effect of the determined Δt parameter on neuronal avalanche analysis is linked to, among others, the determined threshold, as well as to the ongoing inter-event interval distribution, and thus to changes in event rate. Nonetheless, Yu *et al*. indicate that in order to accommodate changes in rate, adaptively changing the applied parameters of the analyses is not properly defined (*i*.*e*., what should be regarded as natural fluctuations in rate as opposed to a robust violation of stationarity, and thus, what should be the division to periods or windows). Moreover, adaptively changing the threshold or alternatively, the duration of the time bins, Δt, constitute two distinctive methodology approaches that alter in a different manner the identity of the collected avalanches (affecting either the above threshold detected events, or their aggregation in-between quiescent periods of at least Δt duration). Yet, there is no clear theoretical rationale to favor adaptively changing one over the other. Therefore, as also claimed by Yu at el., either approach cannot account as a perfect “solution” to temporal changes in rate. Furthermore, as proposed here, adapting the analysis parameters may mask valuable information about the underlying dynamics, such as changes in network excitability and degree of synchrony, which are carried by the changes in the neuronal avalanche metrics.

As experimentally shown before, the parameters Δt and threshold affect the power-law exponent in a predicted manner^11,19,24^. This was also demonstrated in this manuscript (Figs 5 and 6). In agreement with our findings, Yu *et al*. also reported that for all periods, characterized by different event rate, the distributions remained close to power-laws even at a fixed threshold and bin duration, Δt. Therefore, even if one assumes that these changes do not reflect changes in dynamical regimes, the subsequent changes in power-law exponents may simply serve as an easier to apply alternative than adaptively changing a parameter that govern the analysis (Δt or threshold). Another advantage of fixed threshold and bin duration relates to applying the analysis across trials and subjects. This group analysis requires a unified time axis, and hence would not have been possible if the bin sizes were adapted according to the corresponding temporal profile of the event rate for each individual subject. Grouping of cascades follows the spirit of other techniques commonly applied within the cognitive research field. The choice of a temporal scale (Δt), and hence the temporal resolution of evolving cascades, is related to the ratio between the average spacing among sensors and carries an implicit assumption regarding the propagation velocity between sites triggering events. A fixed Δt assumes relatively small variations in the propagation velocity, whereas adaptively decreasing Δt when the event rate increases, implies an increased propagation velocity. Altering Δt with changes in event rate effectively maintains the characteristic temporal scale to be the momentary time between one event to another. However, from a physiological point of view it is not clear why would stimulus-evoked activity propagate faster than ongoing non-evoked brain activity. A fixed threshold may lead to over- or under- estimation of the number of identified events (“noise” in the determination of what should count as an event), whereas adaptively changing the threshold may mask genuine changes in excitability and synchrony, which were previously shown to be associated with the proximity to critical dynamics^6,25,34–36^. Either choice may not be optimal. We argue that fixed analysis parameters, determined from ongoing (or long periods ~1 sec of) brain activity more faithfully portray the underlying dynamics, and that the avalanches obtained under fixed threshold and Δt seem to correctly capture the changing aspects of stimulus evoked activity (Figs 1C,D, 2, 3D,E and 4C,D). We point out that future research may strive to define a cascade by relying on intra-avalanche dynamics.

Variations in power-law exponent were previously shown to relate to altered excitation and inhibition balance, e.g. in sleep deprivation^37,38^ and in epilepsy^25,39^. Focal (or partial) epilepsy is characterized by abnormal activation of hyperexcitable and hypersynchronous localized networks^40,41^. And indeed, zooming on the epileptiform discharges themselves resulted in deviation from scale-free statistics, displaying higher probability than expected from a power-law of a stereotypical avalanche size as well as avalanche spatial dispersion that highlights the epileptic zone^25^. Nonetheless, scale-free behavior is maintained for the ongoing brain activity of epilepsy, which is characterized by shallower power-law exponents than healthy population (*i*.*e*., including not only the discharges themselves but also short time intervals surrounding the discharges, resulted in scale-free avalanche size distributions, yet, with a shallower power-law exponent than for long duration ongoing brain activity of the same epilepsy patients). It seems that even in epilepsy, most of the time and at most locations the epileptic brain follows scale-free dynamics. This supports the claim that scale-free behavior is a robust feature of brain dynamics, even in the pathological state and that following a fixed threshold and bin duration procedure can reflect important changes in the dynamics by a shift in the power-law exponents.

Stimulus-evoked activity involves activations of extensive networks encompassing many brain regions, which is moreover smeared in time by between-trial variability. This may aid to explain the scale-free nature of stimulus-evoked activity, even during short time intervals, as accordingly, and in contrast to the pathological activations of epileptic neural networks, there should be no particular avalanche sizes of excessive probability than predicted by a power-law distribution. Accordingly, the coordinated activity of evoked response across trials does not deviate from a scale free nature, while only by zooming on local or temporally precise grouped activity (bypassing background ongoing activity) this could be the case. It is interesting to compare with another study, which investigated the effect of stimuli on the scale free 1/f component of the spectrum: Podvalny *et al*. 2015 found a change in the power-law exponent of the 1/f component (shallower distributions)^28^. Moreover, at particularly highly activated electrodes, they show a deviation from scale-free behavior associated with a band-limited power rise (a “bump”)^28^. According to the proposed view, across significant intervals of time (~1 sec) there will be compensating mechanisms leading to an overall highly similar dynamics as the dynamics found in rest (Fig. 1A,E and^17^). Nevertheless, at the resolution of short time-intervals of stimulus-evoked activity, the operating point of the dynamics may drift within an extended critical-like region (Griffiths phase)^21–23^, thus, remaining scale-free. The shifts within the stretched critical range correspond to the changes in power-law exponents. Throughout stimulus-evoked activity the branching parameter, σ, which represents the neural gain, deviates from its resting-state value near 1 (Fig. 1B,D and^17^). As the change in power-law exponents, these deviations may also reflect fluctuations in the proximity to the critical state. Nonetheless, if indeed the system is not situated in the vicinity of a single critical point, but rather characterized by “wandering around” an extended “region of criticality”, the mean-field branching process model may not be able to provide an accurate description of the dynamics at the resolution of these short periods within stimulus-evoked activity.

Additionally, it is not obvious whether stimulus-evoked activity should be assigned to the same universality class as resting-state activity, or rather to different ones, which would imply a different set of critical exponents. Mainly, variations in power-law exponents may indicate variations in the associated universality classes. In the stimulus-evoked scenario, the neuronal system is exposed to sensory input that may drive the system. Recent theoretical works examining the influence of external input on neuronal avalanches supports alterations of the critical exponents^14,15,42^. The introduction of inputs is shown to surprisingly preserve the power-law scaling, at various input intensities, while the deviations from the branching process exponents align with avalanche merging (or “gluing”) due to partial loss of separation of time-scale, resulting in smaller power-law exponents, as larger (“glued-together”) cascades become more probable^14^. According to this view, the continuously varying exponent values across time may result from the sensory input driving different initial seeds to form cascades that can merge, leading to larger avalanches and smaller exponents^15^. Importantly, if multiple seeds are involved, adapting the time bin duration, Δt, according to the rate of supra-threshold events will not suffice to untangle parallel-existing avalanches of different origins^43^ (*i*.*e*., in practice the event rate is determined by collapsing all sensors to a single time-trace^11^, thus inherently assuming separation of time scale). Here, the evoked processing of visual stimuli may rely on dynamical changes in effective connectivity at multiple sites, leading to a mean-field branching process description being less adequate. Specifically, spatial effects and effective strength of connections were shown to affect critical exponents^12,44^.

Overall, the presented results support an intermixed nature of the power-law exponents and branching parameters with the parameters that govern the analyses, yet, alongside a robust scale free behavior. When maintaining the parameters that govern the analysis fixed, neuronal avalanches were shown to be able to capture profound changes in the underlying dynamics. The exemplar dynamics of stimulus-evoked response presented in this study, weakens the claim for adapting the parameters of the analyses to follow changes in event rate. Still, constant parameters may not lead to accurately identifying neuronal avalanches as evolving (directed and separated) cascades. A new definition of an avalanche, based on intra-avalanche dynamics, may be a desired goal. Moreover, the time-varying power-law exponent and branching parameter values, may not only reflect the complex underlying dynamics of stimulus-evoked activity, but may also suggest deviations from the description offered by the mean field branching process model, and the existence of different universality classes for different cognitive states, such as resting and evoked activity.

We suggest that different periods within stimulus evoked activity correlate with shifts in an extended critical-like region (Griffiths phase)^21^, which manifest as changes in the corresponding power-law exponents and branching parameters. Taking a broader view, the mean field branching process model may provide relatively accurate description for extended periods and spatial domains, as there are compensating mechanisms involved. Yet, at high temporal or spatial resolutions, there are instantaneous changes in dynamical factors, such as excitability, synchrony and the coordination of activity (effective connectivity) that may lead to drifts in the operating point of the corresponding neural system. We suggest that small drifts will result in scale-free statistics, whereas increased deviations will eventually manifest in breakdown of scale-free statistics.

## Methods

### Experimental procedure and data acquisition

Stimulus-evoked and resting state brain activities were recorded from healthy human subjects (n = 21, age = 22.64 ± 3.03 years) in the MEG facility at the EMBI Unit, Bar-Ilan University, Israel. The study was approved by the Bar-Ilan University ethics committee, in accordance with the relevant guidelines and regulations. The participants gave their informed consent and were financially compensated for their effort. Each subject was recorded during 4 minutes of rest, followed by 10 stimulus-presentation blocks, and 4 additional minutes of rest. During rest, participants were instructed to fixate their eyes on a fixation cross at the center of a black screen. The task stimuli were gray-scale pictures of human faces displaying various emotional expressions and vertical head postures. Each stimulus was presented for 1000 ms with inter stimulus intervals varying between 1,300 and 1,700 ms. Participants completed an oddball gender detection task, in which they were instructed to press a button when a rarely presented female face appeared on the screen (16.67% of the trials). This procedure ensured that all analyzed trials, which consisted of male faces, were task-irrelevant. For additional information, please see^17^.

Neuromagnetic brain activities were recorded with a whole-head, 248-channel magnetometer array (4-D Neuroimaging, Magnes 3600 WH) in a dimly-lit magnetically-shielded room, as participants laid supine. In order to rule out head movements throughout the recordings, head localization measurements were performed before and after each experiment [head position and shape were determined by Pollhemus FASTTRAK digitizer and five coils attached to the participant’s head, measuring position relative to the MEG sensors]. Stimuli were back projected on a screen placed in front of the subjects, by a video projector situated outside the room. E-prime 2.0 (Psychology Software Tools Inc.) was used for experimental control. Participants pressed a button using their right index finger on a response box (LUMItouch photon control) each time a female was presented. The MEG was recorded at a sampling rate of 1017.25 Hz and analog band-pass filtered online at 0.1–400 Hz. Reference coils were used to remove environmental noise. Accelerometers (Bruel and Kjaer) attached to the gantry were used to remove vibration noise. The 50-Hz signal from the power outlet was recorded by an additional channel and the average power-line response to a power cycle was subtracted from every MEG sensor^45^.

### Data analysis

Data processing and analysis were performed using MATLAB 2014a (Mathworks, Andover, MA) and Fieldtrip open-source toolbox for Advanced MEG Analysis^46^.

#### Cleaning and preprocessing

MEG recordings were first cleaned for line frequency, building vibration and heartbeats artifacts with an in-house open-source software^45^. Rest data were segmented to include 1 sec intervals with additional head and tail of 0.4 sec (the 0.4 intervals overlapped with prior and post epochs, but were later cut from analysis). Stimulus-evoked data were segmented to include the 1 sec trials as well as an additional 0.2 pre-trial interval and head and tail of 0.4 sec (as in rest the 0.4 intervals were later cut from analysis). All epoched-data were band-pass filtered offline between 0.8 and 80 Hz. Epochs containing a false-positive response or contaminated by muscle or jump (in the MEG sensors) artifacts were discarded. One mal-functioning MEG sensor (A41) was discarded from all analyses. Independent component analysis (ICA) was performed on the remaining data^47^, to ensure the removal of all eye-movements, blinks and leftover heartbeats artifacts. ICA components reflecting such artifacts, as determined by visual inspection of the 2D scalp maps and time course of that ICA component, were rejected and remaining components were used to reconstruct the data.

To examine bandlimited data, two-pass finite-duration impulse response (FIR) band-pass filter were applied on the whole trials, in accordance with the clinical frequency bands: theta 4–8 Hz; alpha 8–13 Hz; beta 13–30 Hz; gamma 30–80 Hz^48^. FIR filters were of order 3 times the rounded ratio between the sampling frequency and the lower bound of the bandpass frequencies (lowest frequency). This was chosen in order to identify the encompassing oscillations while also restricting the degree of temporal integration caused by the filter.

#### Avalanche analyses

Signal discretization: The signal from each sensor was z-scored by subtracting its mean and dividing by the SD. The mean for each sensor was calculated over the experimental periods of rest, stimulus-evoked and fixation-evoked of each specific subject. Then, positive and negative excursions beyond a chosen threshold for each sensor were identified. A single event was determined per excursion at the most extreme value (*i*.*e*., maximum for positive excursions and minimum for negative excursions). Band-specific avalanche analyses were carried out by subtracting the band-specific mean and dividing by the band-specific SD.

Cascade-size distributions and power law statistics: The time series of events obtained from each sensor for each epoch was individually discretized with time bins of duration Δt. The temporal resolution of the analysis Δt is a multiplication by an integer of Δt_min_ = 0.983 msec (wheras Δt_min_ is the inverse of the data acquisition sampling rate). A cascade is defined as a continuous sequence of time bins in which there is an event on any sensor, ending with a time bin with no events on any sensor. The number of events on all sensors in a cascade is defined as the cascade size.

According to the theory of critical branching processes, power-law behavior is predicted at the critical state (Harris T. E. 2002). The fit of the avalanche size distributions to a power law was analyzed as described previously^49,50^. The candidate distributions were power law and exponential distributions: both characterized by a single parameter (degree of freedom), and lognormal, stretched exponential and exponentially truncated power law distributions: all three characterized by two parameters.

Power laws were modeled as follows:

1 $${P}_{\alpha }(x)=\{\begin{array}{cc}{C}_{\alpha }{x}^{\alpha } & {x}_{min}\le x\le {x}_{max}\\ 0 & otherwise\end{array}$$

Exponential distributions were modeled as follows:

2 $${P}_{\lambda }(x)=\{\begin{array}{cc}{C}_{\lambda }{e}^{-\lambda x} & {x}_{min}\le x\le {x}_{max}\\ 0 & otherwise\end{array}$$

Lognormal functions were modeled as follows:

3 $${P}_{\mu ,\sigma }(x)=\{\begin{array}{cc}\frac{{C}_{\mu ,\sigma }}{\sqrt{2}\pi \sigma x}exp[-\frac{1}{2}{(\frac{\mathrm{ln}x-\mu }{\sigma })}^{2}]\, & {x}_{min}\le x\le {x}_{max}\\ 0 & otherwise\end{array}$$

Stretched exponential functions were modeled as follows:

4 $${P}_{\beta ,\lambda }(x)=\{\begin{array}{cc}{C}_{\beta ,\lambda }{e}^{-{(\frac{x}{\lambda })}^{\beta }} & {x}_{min}\le x\le {x}_{max}\\ 0 & otherwise\end{array}$$

Exponentially truncated power laws were modeled as follows:

5 $${P}_{\alpha ,\lambda }(x)=\{\begin{array}{cc}{C}_{\alpha ,\lambda }{x}^{\alpha }{e}^{-\lambda x} & {x}_{min}\le x\le {x}_{max}\\ 0 & otherwise\end{array}$$

where Cα, C_λ_, C_μ,σ_, C_β,λ_ and C_α,λ_ are normalization factors, and the parameters x_min_ and x_max_ were set to include all observed avalanches (x_min_ = 1 and x_max_ = 320).

A maximum likelihood estimation was applied directly to the sample of avalanche sizes, and not on the distributions. Accordingly, the obtained estimations are more sensitive to sizes of higher probabilities (small avalanches). In comparison, direct regression of the distributions would have assigned the same weight to avalanches of low probability as to those with high probability and thus would have been more affected by the predicted cutoff of the power law around system size (i.e., number of sensors in the array).

Assuming independence of avalanche sizes and a sample of *n* avalanches, the likelihood of a sample of avalanche sizes given the power law and exponential models given a parameter α or λ is:

6 $$L(param|x)=\,\mathop{\prod }\limits_{i=1}^{n}\,{P}_{param}({x}_{i})$$

While the log-likelihood is given by:

7 $$l(param|x)=\,\mathop{\sum }\limits_{i=1}^{n}\,\mathrm{ln}\,({P}_{param}({x}_{i}))$$

The best fit parameters for the power law and exponential distributions ($\hat{\alpha }$ and $\hat{\lambda }$) were calculated by maximizing the log-likelihood as a function of the parameter. To determine whether a power law or an exponential distribution was a better fit to the data, the log of the likelihood-ratio (LLR) was taken with the best fit parameters as follows:

8 $$LLR({x}^{(n)})=l(\hat{\alpha }|{x}^{(n)})-l(\hat{\lambda }|{x}^{(n)})$$

Thus, a positive LLR indicates that the power law model is more likely, while a negative LLR indicates that the exponential model is more likely; for an LLR of zero, neither distribution is more likely. To determine whether the LLR was significantly different from zero, the p value of the LLR was calculated as follows:

9 $$p=erfc(\frac{LLR}{\sqrt{2n{\sigma }^{2}}})$$

where: ${\sigma }^{2}=\frac{1}{n}\mathop{\sum }\limits_{i=1}^{n}\,{[(l(\alpha |{x}_{i})-\overline{{l}_{a}})-(l(\lambda |{x}_{i})-\overline{{l}_{\lambda }})]}^{2}$ with $\overline{{l}_{a}}=l(\alpha |{x}^{(n)})/n$ and $\overline{{l}_{\lambda }}=l(\lambda |{x}^{(n)})/n$.

The LLR for the comparison between each pair of the distributions: exponentially truncated power law, stretched exponential and lognormal distributions can be calculated analogously (Eqs 8 and 9). Since these distributions have an additional degree of freedom as compared to the power law and exponential models, the LLR test would have been difficult to interpret if models with different number of degrees of freedoms were intermixingly compared. Thus, only models with the same number of degree of freedoms were tested against each other^50^. Throughout this article, the reported power law exponent, α, is the one fitted to the simple power law model (Eq. 1), and not to the exponentially truncated power law (Eq. 5). The exponential decay multiplication factor in the exponentially truncated power law model is conventionally inserted to capture the cut-off of the power law behavior at approximately system size (in our case, size of the sensor array). However, it affects the entire range, thus, distorting the power law exponent itself. Therefore, the exponentially truncated power law model is suboptimal. Nonetheless, it is important to point out that the same trend of results was obtained for the power law exponents taken from the exponentially truncated power law as for the results for the power-law exponent in the naïve power-law model.

Notably, Clauset *et al*.^49^ suggest to test the goodness of fit between the data and the power law model (if p-value is smaller than 0.1, the power-law hypothesis is rejected). However, Klaus *et al*.^50^ who statistically analyzed the fit to a power-law model in neuronal avalanches, refer to the test offered by Clauset *et al*.^49^, and claim that the offered test is misleading for large enough sample size. Klaus *et al*.^50^ claim that even a small (practically negligible) deviation from a power law will result in the rejection of the power-law hypothesis for most empirical systems in the large sample size regime. They show that the p-value depends on the sample size of the empirical distribution. The distributions in our manuscript are based on tens of thousands of samples – the same sample size regime that the results of Clauset *et al*.^49^ goodness-of-fit test are claimed by Klaus *et al*.^50^ to be misguiding.

The branching parameter: The branching parameter σ was estimated by calculating the ratio of the number of events in the second time bin of a cascade to that in the first time bin. This ratio was averaged over all cascades for each subject and for each experiment part, with no exclusion criteria^11^ as follows:

10 $$\sigma =\frac{1}{{N}_{av}}\mathop{\sum }\limits_{k=1}^{{N}_{av}}\,\frac{{n}_{events}(2nd\,bin\,of\,k^{\prime} th\,avalanche)}{{n}_{events}(1st\,bin\,of\,k^{\prime} th\,avalanche)}$$

where N_av_ is the total number of avalanches in the particular dataset and n_events_ represents the number of events in a particular bin.

Group avalanche analyses (across subjects) were carried out by accumulating all identified cascades from all preprocessed segments of all subjects, while cascades from rest and stimulus-evoke were collected separately. This enabled focusing on shorter time intervals (within stimulus-evoked trials or rest) by separately accumulating the identified cascades at specific intervals (*i*.*e*., at sequential 80 msec intervals).

#### Spectral analyses

Time-frequency representations of rest and stimulus-evoked activities were performed by effectively convolving the data with complex wavelets (applying Fieldtrip’s “mtmconvol”: multitaper-method convolution). Each complex wavelet was constructed by time-point wise multiplication of the cosine (real) and sine (imaginary) components at the specific frequency with a tapering function. Here, Hann tapers were chosen for the frequencies between 4 till 80 Hz. The lengths of the sliding time windows were set to include 3 cycles per time window. This resulted in power-spectra estimates as a function of time^51^. In order to obtain “ERF time-frequency power” estimates, first the ERF was computed by averaging across subjects and trials, aligned to stimulus onset: t = 0 (or equivalent rest epochs), and then calculating its time-frequency representation. Alternatively, in order to obtain “total power” spectra across time, first the time-frequency decomposition of each trial was calculated and then averaging those results from all subjects and trials together. Given the short intervals of interest (≪~1 sec trials), enabling the sampling of only a small number of cycles, delta frequency band was not investigated in this research.
